# Research of real-time corn yield monitoring system with DNN-based prediction model

**DOI:** 10.3389/fpls.2024.1453823

**Published:** 2024-08-27

**Authors:** Chaojie Yin, Qi Zhang, Xu Mao, Du Chen, Shengcao Huang, Yutong Li

**Affiliations:** ^1^ College of Engineering, China Agricultural University, Beijing, China; ^2^ Beijing Key Laboratory of Optimal Design of Modern Agricultural Equipment, China Agricultural University, Beijing, China; ^3^ National Innovation Centre for Agricultural Machinery and Equipment, Luoyang, China; ^4^ Beidahuang Agricultural Service Group Heilongjiang Agricultural Machinery Service Co., Harbin, China

**Keywords:** corn combine harvester, monitoring system, EDEM, machine learning, yield prediction

## Abstract

The real-time monitoring of corn yield by a combine harvester is a critical data source for constructing the yield histogram, which significantly benefits precision management and decision-making in modern precision agriculture. While widely used, the current photoelectric sensor-based yield monitoring method has limitations. It detects the corn height on each scraper and calculates the yield through a geometric formula. However, it neglects the noticeable difference in the corn stacking patterns affected by factors such as feeding volume, terrain, and driving speed. This oversight often results in low accuracy and poor stability in the prediction of corn yield, highlighting the need for a more advanced approach. To resolve this, we employ EDEM discrete element simulation to demonstrate the large difference of corn stacking patterns on the scraper of the elevator corresponding to feeding volume. Then, we develop a real-time monitoring system on our self-developed double elevator testing rig for carrying out a composite dataset for training three machine learning algorithm-based models, namely Deep Neural Networks (DNN), Gradient Boosting Machine (GBM), and Random Forest (RF). Importantly, these models have undergone rigorous validation under various feeding volumes, ensuring their robustness and reliability. The auxiliary elevator speed is meticulously set at 150r/min, 225r/min, and 450r/min, providing a comprehensive performance assessment. The results denote that the DNN model performs best and is stable, with a coefficient of determination (R^2^) of 0.998, root mean square error (RMSE) of 0.526, and mean absolute error (MAE) of 0.425. The paper also performs field experiments to test the proposed three prediction models and the system. The results also denote the DNN-based prediction model’s best performance for the lowest relative error of 2.29% and the highest average accuracy of 97.85%. Consequently, the proposed real-time corn yield monitoring system achieves high accuracy and reliability for the combine harvester applications.

## 1 Introduction

*Precision agriculture* is a modern management approach supported by information technology that relies on multidimensional and accurate data to provide rational management decisions (Wang, 1999; Zhao et al., 2003; Reinke et al., 2011). Instantaneous grain flow rate is key to generating an accurate yield map. Each block of information on the yield map is derived from the instantaneous grain flow rate data. Only precise instantaneous flow rate information can ensure the accuracy of the yield map. Therefore, real-time yield information can generate a yield map, enabling agricultural producers better to understand the dynamics of field variability and crop growth to adjust the crop management strategies for the next planting round (Mistry and Bora, 2019). As the world’s most important and productive crop, corn accounted for 35% of the world’s total crop in 2018. It is also one of the three significant crops in China. Hence, it is essential to implement real-time yield monitoring during corn harvesting.

Corn yield monitoring can be achieved by different methods, including sample estimation, remote sensing, Internet of Things (IoT) technology, and direct measurement on a combine harvester (Zhang, 2003; Veal et al., 2010; Zhu et al., 2018; Omasa et al., 2022; Yin et al., 2022) in which sample estimation highly depends on the representative and sampling frequency. Unfortunately, the accuracy can hardly be assured. Remote sensing technology restricts the accuracy due to cloud coverage, image resolution, and algorithms. The Internet of Things (IoT) has a high cost for deploying abundant sensors and equipment, resulting in complicated data processing. None of the abovementioned methods is feasible for monitoring the corn yield in real-time. Hence, this study aims to develop direct measurement methods deployed in combine harvesters to realize real-time yield monitoring during harvesting. Currently, the direct measurement methods used in combine harvesters are categorized into dynamic weighing, impulsive, volumetric, and ray measurements (Wang et al., 2021). More research has been carried out based on impulsive and volumetric measurement methods. Chen et al. (Chen et al., 2010) designed a double-plate differential impulsive grain flow sensor and demonstrated good measurement accuracy in field trials. Zhou et al. (Zhou et al., 2006) designed a parallel beam impulsive grain mass flow sensor as the core of the yield measurement system. However, the pulse beam of the impulse sensor is relatively narrow, and the sensor installation is complex, which hinders the method’s practical application under complex field and working conditions. Fu et al. (Fu et al., 2017) used a diffuse reflective photoelectric sensor to measure the pulse signal, established a segmented grain yield conversion model, and further outcome a measurement system. Jin et al. (Chengqian et al., 2022) adopted a pair of opposed photoelectric sensors to obtain the data and predict the yield upon the duty cycle and a yield measurement model, reporting a maximum error of 3.83% in field trials. All the above studies provide an essential base for utilizing direct measurement methods while developing a real-time monitoring system for yield prediction in field scenarios.

The following problems still exist in yield measurement with photoelectric sensors: 1) pre-experimentation before each test is necessary for calibration, but performing it in fields can be complex, cumbersome, and lacks maneuverability; 2) prediction with a scraper-geometric formula is inaccurate since the corn stacking pattern during lifting on a scraper can be varied corresponding to multiple factors including feeding volume, driving speed, and slope of elevator and terrain.

In recent years, machine learning, as a core of artificial intelligence (Shaikh et al., 2022), has been increasingly used in field applications (Zhang, 2021), producing from a large amount of data and making decisions to promote the efficiency of agricultural production (Van Klompenburg et al., 2020). Liu et al. (Liu et al., 2019) utilized the random forest algorithm to construct a multivariate combination model for the regression and prediction of winter wheat yield. Zhou et al. (Zhou et al., 2023) studied four machine learning algorithms, multiple linear regression, random forest, adaptive augmentation model, and artificial neural network, to build a model for predicting soybean yield and weighing the influencing factors.

This paper aims to study yield monitoring using machine learning methods. Through EDEM discrete element modeling and simulation, it simulates the corn kernel elevating process under different feed rates and verifies that the accumulation pattern of corn kernels on the scraper is not fixed (Owen and Cleary, 2009). As a novel approach, we first built an indoor testing rig to mimic the corn elevating and measure the corn volume rate in real-time with our developed data acquisition system based on the low pulse-width measuring method. We designed tests for a composite situation and trained yield prediction models using three machine learning algorithms: deep neural network (DNN), gradient boosting machine (GBM), and random forest (RF). Comparative indoor and in-field experiments were then conducted to verify the feasibility and accuracy of the proposed corn yield monitoring system and determine the most appropriate prediction model.

## 2 Materials and methods

### 2.1 Mechanism of stacked corn height acquisition

**Figure 1:**
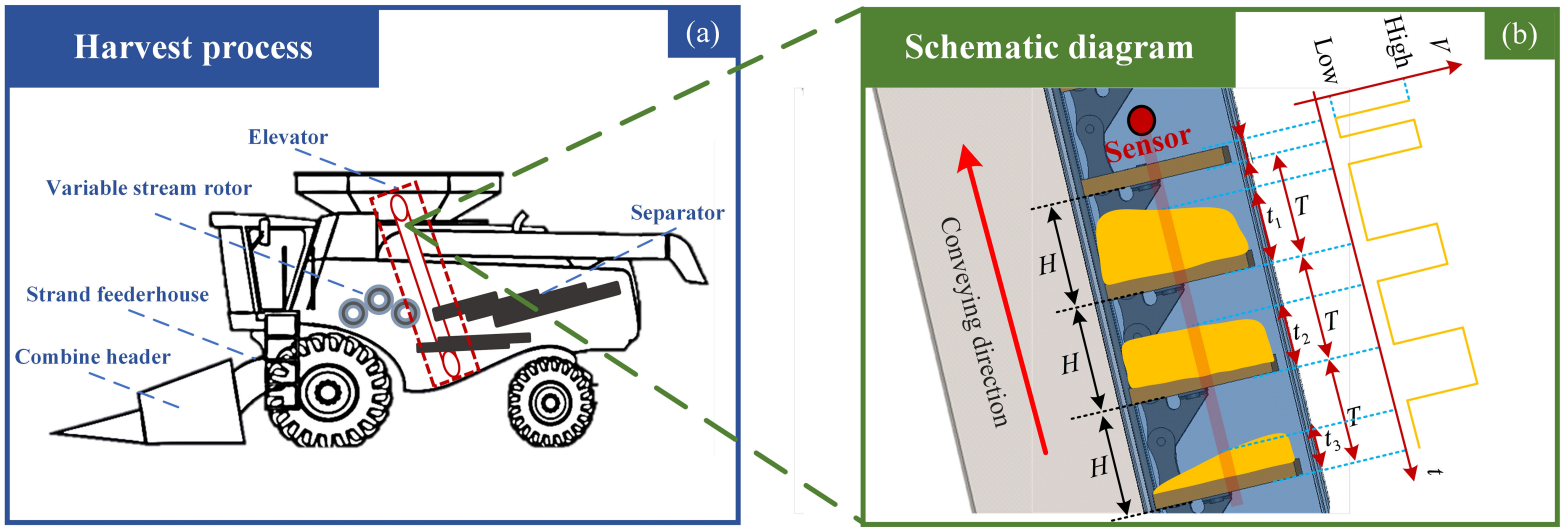
Schematic diagram of **(A)** harvesting process of a combine harvester, and **(B)** developing a system for monitoring the harvester's working process.

A photoelectric-based yield monitoring system often deploys sensors near the top of a combine’s elevator, as shown in **Figure 1A**. The system mainly detects the height of the stacked corn on each scraper in the elevator and predicts the yield by a model with its schematic diagram shown in **Figure 1B**. In a harvester, the auger and elevator jointly operate to convey the corn to the harvester’s tank. The auger horizontally transports the corn while the elevator transports the corn to the tank in an inclined or quasi-vertical direction. The system uses an opposed beam-type photoelectric sensor, whose one end emits an infrared light beam in a certain area while the other outputs a variant signal to inform whether or not an object blocks the beam. The NPN-type sensor outputs a high level when the beam is fully received and a low level when the beam is blocked. The sensor is installed at the top of the elevator because kernels on the internal scraper may fall off during the lifting process during the transfer of corn kernels by the elevator. Installing the sensor at the bottom or middle of the elevator would result in significant errors. A proper sensor position can avoid signal interference and measurement errors caused by corn drops. The mechanism of the corn yield monitoring system in the present paper is as follows: in non-loading conditions, the shading time by a lifting scraper is *t_0_*, which denotes the time of the sensor’s low pulse width output.

During the harvesting process, the system operates as follows: the time interval between the two adjacent scrapers is *T*, which refers to two adjacent rising edges of the signal. In the non-loading condition, the idling low pulse width time ratio is *P_0_=t_0_/T*, representing the duty cycle per cycle time. Each scraper’s masking time is *t_1_*, which is the observed low pulse width time passing the scraper and stacked corn. Therefore, the masking ratio is *P_1_=t_1_/T*, denoting the scraper and corn’s time ratio over each cycle. Then, the masking time ratio for corn is extracted by *P_g_* =*P_1_*-*P_0_*, and the stacked corn height is *h*_1_=H*P_g_, where *H* is the height between two adjacent scrapers. We also obtain that,


(1) $$h_{n}=H\left(\frac{t_{n}}{T}-\frac{t_{0}}{T}\right)$$

where, *h_n_* is the staking corn height on the *n*th scraper, and *t_n_* is the masking time of the *n*th scraper.

### 2.2 Harvest yield monitoring system composition

According to the requirements, the study builds the monitoring system upon a pair of photoelectric sensors, a GNSS positioning module, an antenna, a power supply unit, a data acquisition card, and a triple-proof computer. The photoelectric sensor is the Omron E3FA-RN21, and the data acquisition board is the Beijing Simai Kehua’s USB-1252A with 40 ns for the pulse width measuring time. The GNSS module is Beiyun Technology’s high-precision positioning and directional receiver T1, used for obtaining real-time position information of the corn harvester. The power supply unit reduces the 12V to 5V to power the data acquisition card while powering 12V to other equipment. The system uses a triple-proof computer for operation, real-time yield prediction, and other information displays.

**Figure 2:**
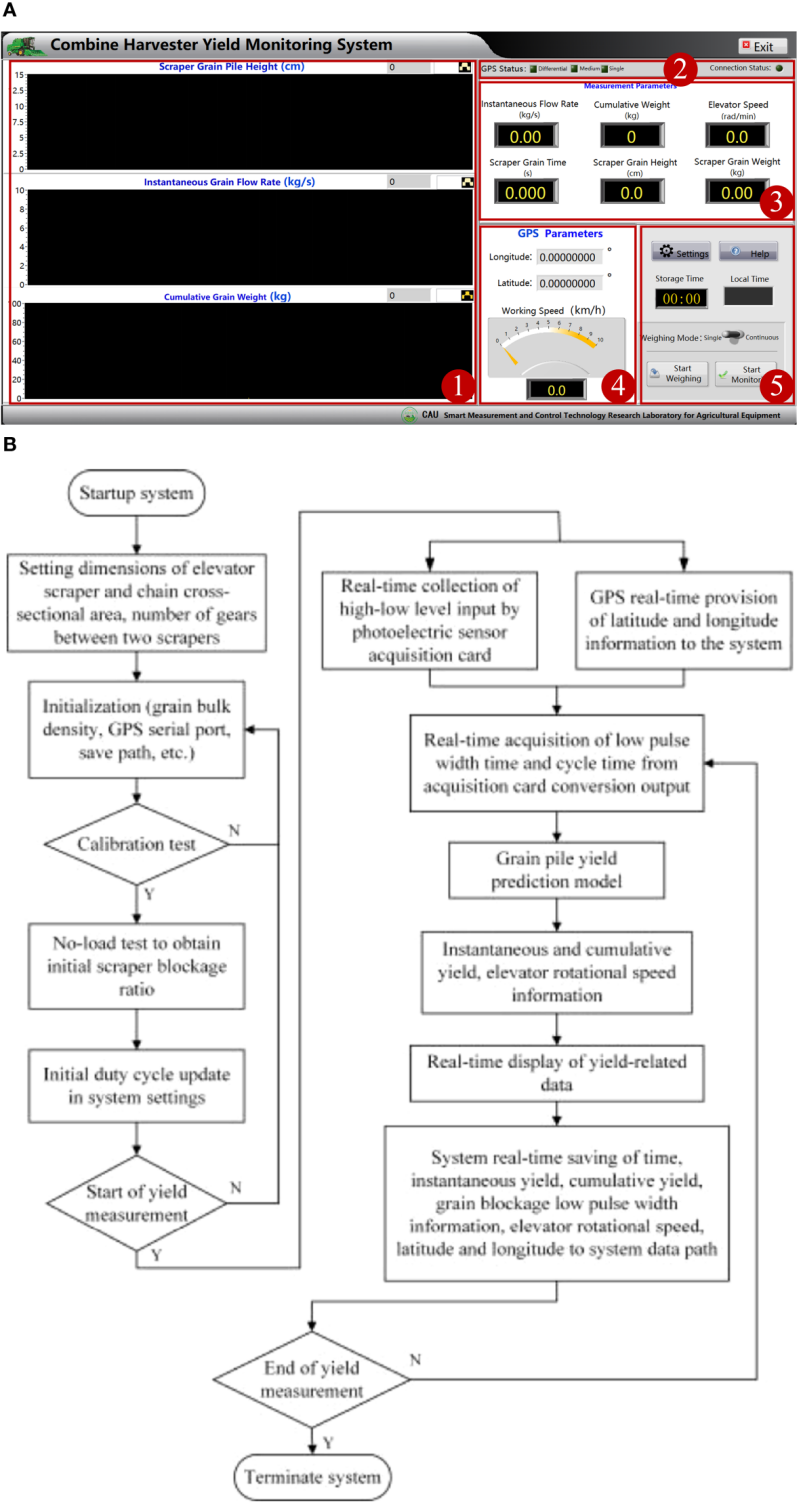
**(A)** System function interface design diagram and **(B)** system function flowchart. 1. Maize yield infographic display module. 2. Device connection status display module. 3. Numerical display module for monitoring parameters. 4. GPS related parameter display module. 5. Function setting module.

The study adopts LabVIEW for system software, which includes parameter and function settings, graphical displays of corn yield, numerical displays of monitoring parameters, GPS-related parameters, and equipment connection status, as shown in **Figure 2A**. A parameter setting is required before starting yield monitoring. The parameter setting configures the chain’s cross-sectional area, the scraper’s length, width, and thickness, the space between neighboring scrapers, and the elevator’s gear numbers.

Afterward, a no-load preprocessing test is required to obtain the combine harvester’s raw scraper shading duty ratio (initial low pulse width time to cycle time ratio) and then input information such as maize weight determination, GPS serial channel, and save path. The function setting configures the corn weight correction factor, GPS serial channel, and directory path. The setting also requires performing a non-loading test to obtain the harvester’s idling low pulse width time ratio. The acquisition board acquires the sensor signals’ high- and low-level variation at a high speed. The board converts signals’ variations into low pulse width and cycle time variables. Then it transmits them to the system to calculate the staking corn height and the masking time for each scraper. The system introduces these parameters and variables into the yield prediction model for outputting the instantaneous corn flow rate and cumulative weight. Lastly, the system saves data, including time, instantaneous yield, cumulative weight, latitude, longitude, and driving speed, in a file following the directory path. **Figure 2B** shows the whole workflow of the corn harvester’s yield monitoring system.

### 2.3 EDEM Simulation of corn elevating

#### 2.3.1 Corn kernel and elevator modeling

**Figure 3:**
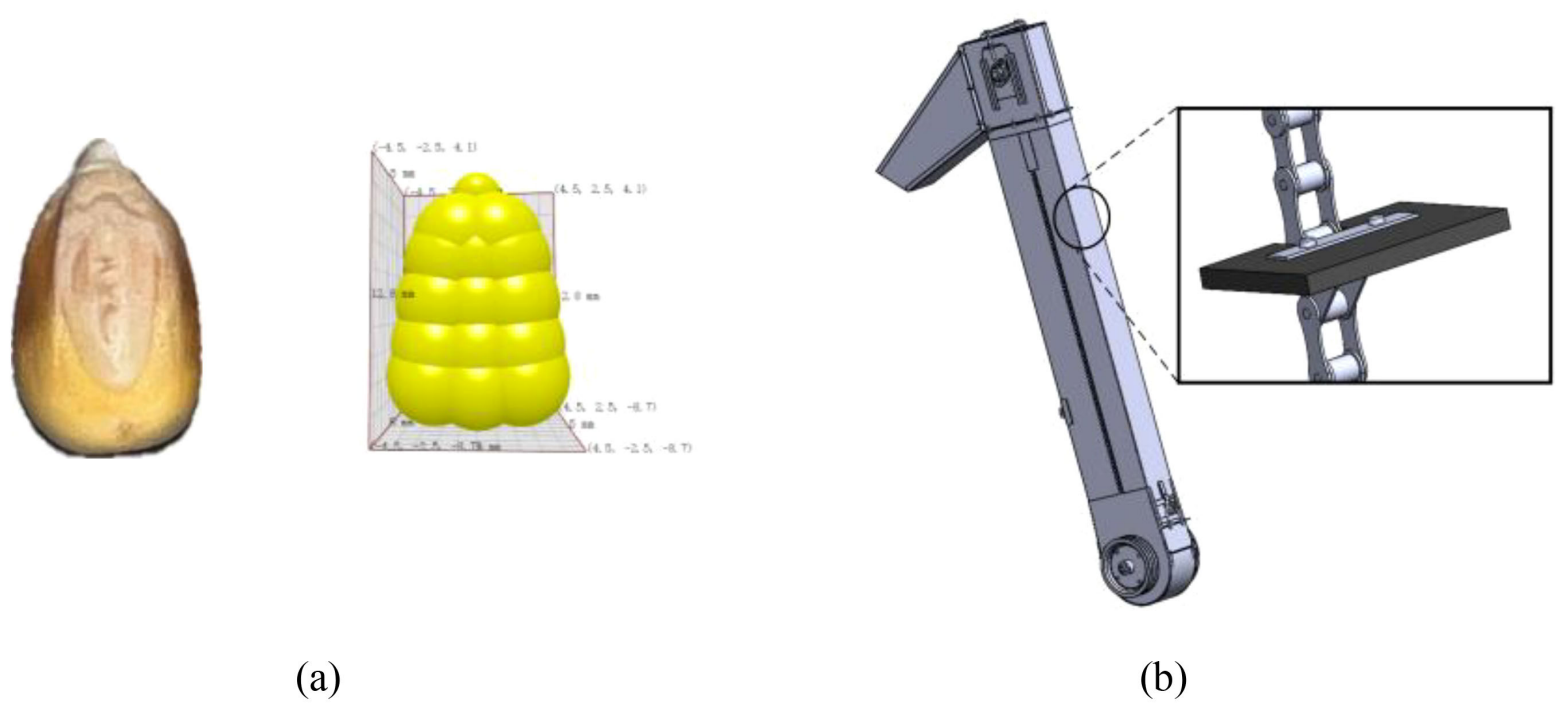
Necessary elements in the EDEM model **(A)** single corn particle model **(B)** elevator model.

Due to the high elevator speed of the combine harvester under field operating conditions, it is difficult to capture accurate observations. Additionally, during the lifting process, corn kernels may fall, making it impossible to directly observe the accumulation of corn kernels through effective means. Therefore, EDEM simulation can be used to observe the changes in corn morphology under different feed rates. To realize this, we adopt EDEM software to simulate and analyze the corn stacking behavior on the elevator scraper associated with different feeding amounts. Firstly, a corn kernel model is constructed in EDEM to represent a physical corn. The study repeats measurements several times and takes the average value, having the three dimensions of a corn kernel as 12.8mm in length, 9mm in width, and 5mm in thickness. The study utilizes 15 small spheres to combine and generate the corn model shown in **Figure 3A**. Next, the elevator is determined to have an equal scale to the actual harvester’s elevator and is built in SolidWorks, as shown in **Figure 3B**. The elevator’s side wall and chain are made of steel, while the scraper is made of rubber. According to the literature (Wang et al., 2016) corn kernels constantly collide with the elevator’s inner wall, chain, and scraper. Thus, the mechanical properties of corn, scraper, and elevator are carefully determined (Liu et al., 2022; Hongmei et al., 2023), as are the collision parameters. **Table 1** demonstrates the properties mentioned above. The collision model used in the simulation is Hertz-Mindlin (no slip), and the friction model is Standard Rolling Friction.

**Table 1:** Individual material and contact parameter settings.

Material	Parameter	Value
Corn kernels	Poisson’s ratio Shear modulus/Pa Density/(kg/m^3^)	0.4 2.17×10^8^ 1250
Steel plate	Poisson’s ratio Shear modulus/Pa Density/(kg/m^3^)	0.3 7.0×10^8^ 7800
Rubber	Poisson’s ratio Shear modulus/Pa Density/(kg/m^3^)	0.45 0.5×10^8^ 1300
Corn kernels with corn kernels	Recovery coefficient Static friction factor Rolling friction factor	0.182 0.420 0.080
Corn kernels with rubber	Recovery coefficient Static friction factor Rolling friction factor	0.500 0.450 0.035
Corn kernels with steel plate	Recovery coefficient Static friction factor Rolling friction factor	0.532 0.482 0.092

#### 2.3.2 Lifting configuration and simulation

**Figure 4:**
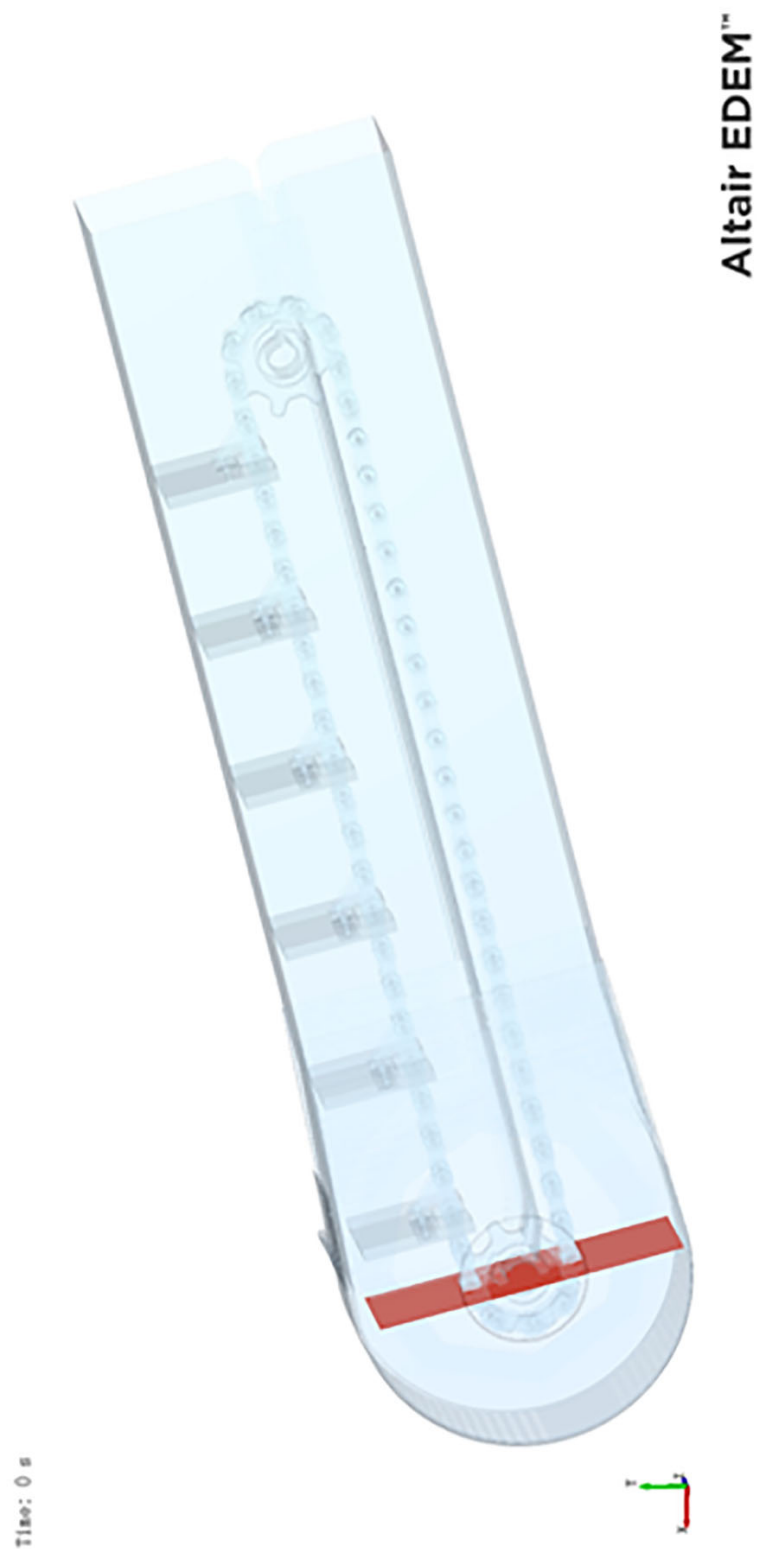
Simplified elevator and bottom built pellet plant plane.

The model only keeps the elevator and discards the auger to reduce the computational cost and guarantee the smoothness of the simulation. The structure’s transparency is set to 0.3 to visualize the animation. Most harvesters’ elevators tilt between 5° and 15°. Such an angle allows corn to flow over the scraper while avoiding uneven stacking or slippage. In this study, the tilt angle *θ* is 15°. The elevator’s cross-sectional area is 300mm in length and 150mm in width. A rectangle of the same size is 150mm from the bottom of the elevator. This position is determined where the auger feeds corn into the elevator. The software uses this rectangular area as the corn plant to generate corn kernels, as displayed in **Figure 4**.

In the harvesting circumstance, the feeding rate or feeding amount varies in terms of corn density and operating parameters. The feeding amount, in turn, dramatically affects the corn stacking pattern on the scraper. The study simulates three conditions regarding low, medium, and high feeding amounts for a comprehensive investigation and analysis. The study utilizes rotary and linear motions for scrapers and chains to lift the corn kernels for the additive motion. The elevator’s rotational speed ranges from 500 to 600r/min, with the linear motion speed being 2.5m/s since the elevator can constantly rotate in a harvester. The elevator model in the simulation is constructed based on the actual elevator of the combine harvester. The corn feed rates required for the simulation under different conditions are calculated by measuring the height between the scrapers in the elevator. After calculation, the initial corn weight of 2 kg, 5 kg, and 8 kg meets the demand to simulate low, medium, and high feed rate scenarios.

#### 2.3.3 Morphological analysis of corn kernel stacking

**Figure 5:**
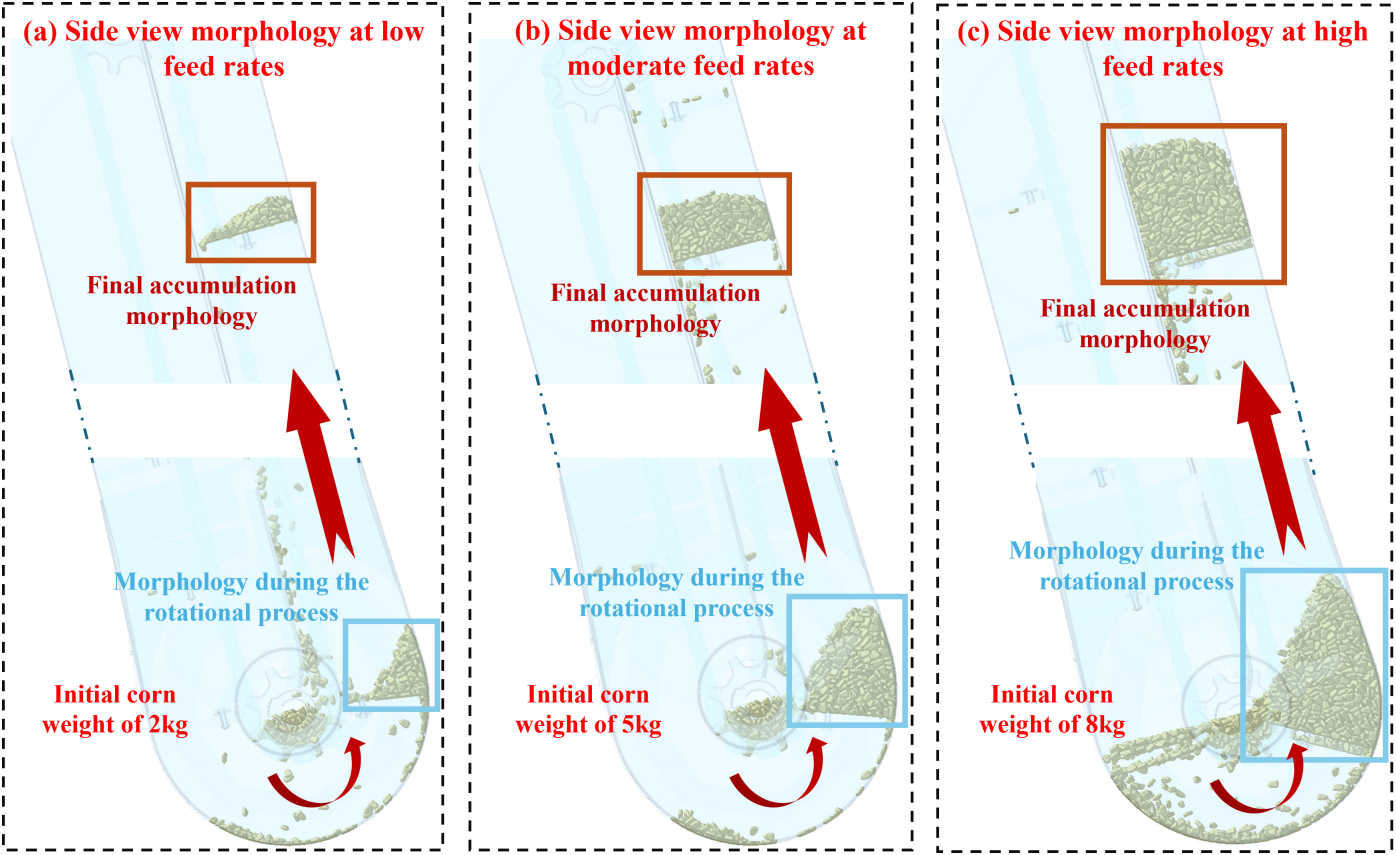
Corn accumulation at different feed rates. **(A)** The side view morphology at low feed rate, **(B)** at moderate feed rate, and **(C)** at high feed rate.

**Figure 5** demonstrates the morphology of corn stacking in terms of different feeding amounts. When the plant imports 2kg of corn kernels, the kernels pile up at the outer end of the scraper while the inner end has less corn. The morphology of stacking corn retains a similar shape while lifting the scraper, indicating a stable stacking process that is not significantly affected by the elevator tilt and gravity. The stable shape of the corn accumulation is observed as a trigonometry. When the plant imports 5kg of corn kernels, the corn stacking on the scraper is centered and piles up at the outer end when the scraper rotates at the bottom of the elevator. However, corn deviates to the inner end during lifting: the elevator tilt and gravity cause the phenomenon. The morphology varies from the light feeding amount, and the final morphology becomes a rectangle. When the plant imports 8kg of corn kernels, the outer end of the scraper is full of corn. When the scraper ascends, the tilt and gravity compress the corn towards the inner end, resulting in higher inner accumulation than the outer. Moreover, the final morphology is a trapezium, and the incline angle tends to parallel the scraper. Based on the analysis, the morphology of corn stacking patterns on the scraper varies via lifting at different feeding amounts. Therefore, it is reasonable that a simple mathematical model only formulated by the scraper geometry will be inaccurate for predicting the yield. Repetitive corrections of such a model are required when the harvester operates at different feeding conditions, which is cumbersome, inconvenient, and lacks maneuverability.

#### 2.3.4 Yield prediction model construction

During the field operation of the combine harvester, the scraper in the elevator conveys the grain into the grain tank. During this process, the height information of corn kernels on the elevator scraper is the most crucial data for reflecting the yield of the combine harvester. This information is also the most effectively and conveniently one to be collected by a system, and can well-illustrate the variation of flow rate and instantaneous yield. In this process, the system is well-designed to collect the variable value of the grain pile height inside the elevator, which best reflects the accumulation of kernels on the scraper at each instance. Deep Neural Network (DNN), Gradient Boosting Machine (GBM), and Random Forest (RF) are trained to construct a yield prediction model under the composite condition. Then, the study tests these models’ performances with three working conditions. The coefficient of determination (R^2^), root mean square error (RMSE), and mean absolute error (MAE) are the model evaluation metrics. R² is chosen to better reflect the goodness of fit of the model, thereby evaluating the overall predictive performance of the system. RMSE and MAE are selected to reflect the average error between the predicted and actual values, allowing for the assessment of the system’s accuracy and precision. The coefficient of determination evaluates the fitting effect of the models, with its value closer to 1 indicating a better model fitting and the overall performance of the system is improved. The root mean square and absolute errors assess the model’s accuracy, whose values closer to 0 indicate more precise predictions and the higher the accuracy and precision of the system.

## 3 Result and discussion

### 3.1 Indoor elevating experiment and yield prediction model

#### 3.1.1 Indoor double-elevator testing rig

**Figure 6:**
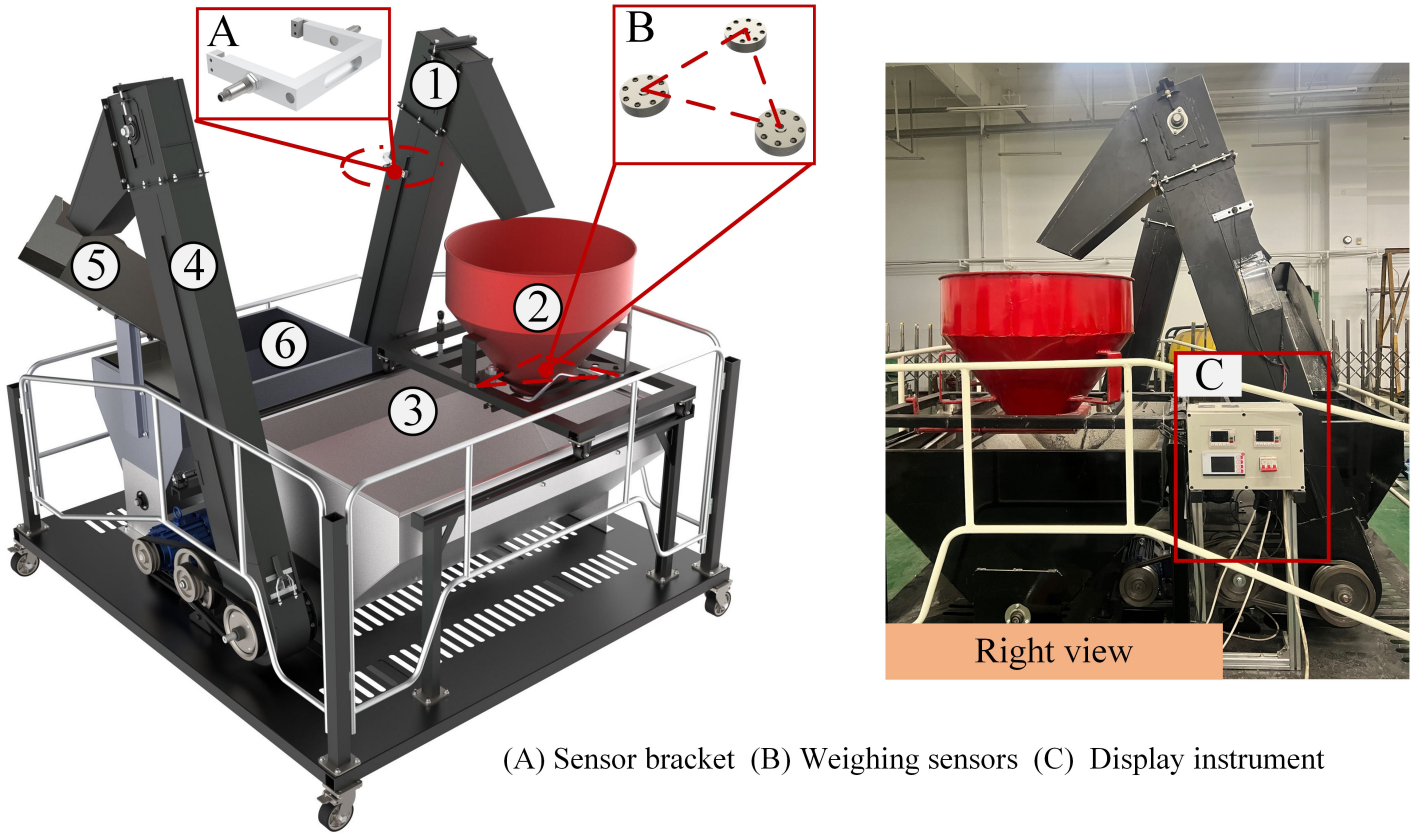
Composition diagram of the test stand. 1. Main elevator. 2. Weighing bucket. 3. Transfer bin. 4. Auxiliary elevator. 5. Sieve. 6. Feeding bin. **(A)** is the sensor bracket installing on the top of elevator, **(B)** shows weighing sensors, and **(C)** is the display instrument.

The experimental bench is designed according to the structure and size of the 4YL-6 (8568) corn harvester’s internal elevator, independently developed by the National Innovation Centre for Agricultural Machinery and Equipment. It mainly consists of six parts: the main elevator, weighing bucket, transfer bin, auxiliary elevator, sieve, and feeding bin, which are capable of completing the functions of maize circulation and real-time weighing. Two DC motors drive the main elevator, auxiliary elevators, and auger. The sensor bracket is used to fix the opposite-type photoelectric sensor on the main elevator, as shown in **Figure 6A**. The weighing bucket with a maximum capacity of 100 kg is designed to accommodate the corn discharged by the elevator. In order to obtain the accurate weight of the harvested corn in real-time, we adopt a three-point measurement method is used for weighing by adding high-precision spoke weighing sensors, as shown in **Figure 6B**. The weighing sensor model is Decent DSLF-102, with a 0-100kg range and a measurement accuracy error of less than 0.05%. As illustrated in **Figure 6C**, the rig installs a frequency converter to control the motor speed, allowing the elevator and auger speed adjustment by varying the frequency of the converter.

#### 3.1.2 Establishment of yield dataset

The study designs the training dataset under a composite working condition and a verification dataset under three independent working conditions, including low, medium, and high feeding amounts. Jin et al. (Chengqian et al., 2022) studied the grain accumulation patterns at different elevator speeds, however, the investigation of the effect of the accumulation patterns under varying feed rates is not obvious. This study proposes using different auxiliary elevator speeds to generate obvious feed rate changes during actual operations, providing a more comprehensive analysis of corn kernel accumulation patterns inside the elevator. In the experiment, the main elevator’s speed is set at a constant 550 r/min. The low feeding amount signifies the auxiliary elevator’s speed of 150 r/min, 225 r/min for the medium, and 450 r/min for the high. The height and maize bucket’s weight information are synchronously collected. During each test, the system collects the height information of the corn pile on the main elevator’s scraping board. The weighing sensor records the yield weight data correspondingly and transmits the data to the system. The composite working condition integrates low, medium, and high feeding amount conditions. When the feeding amount changes from low to high, the data are continuously collected to build the composite dataset. The verification dataset contains two sorts of data while performing three working conditions, respectively. 3.1.3 Preprocessing of raw data.

**Figure 7:**
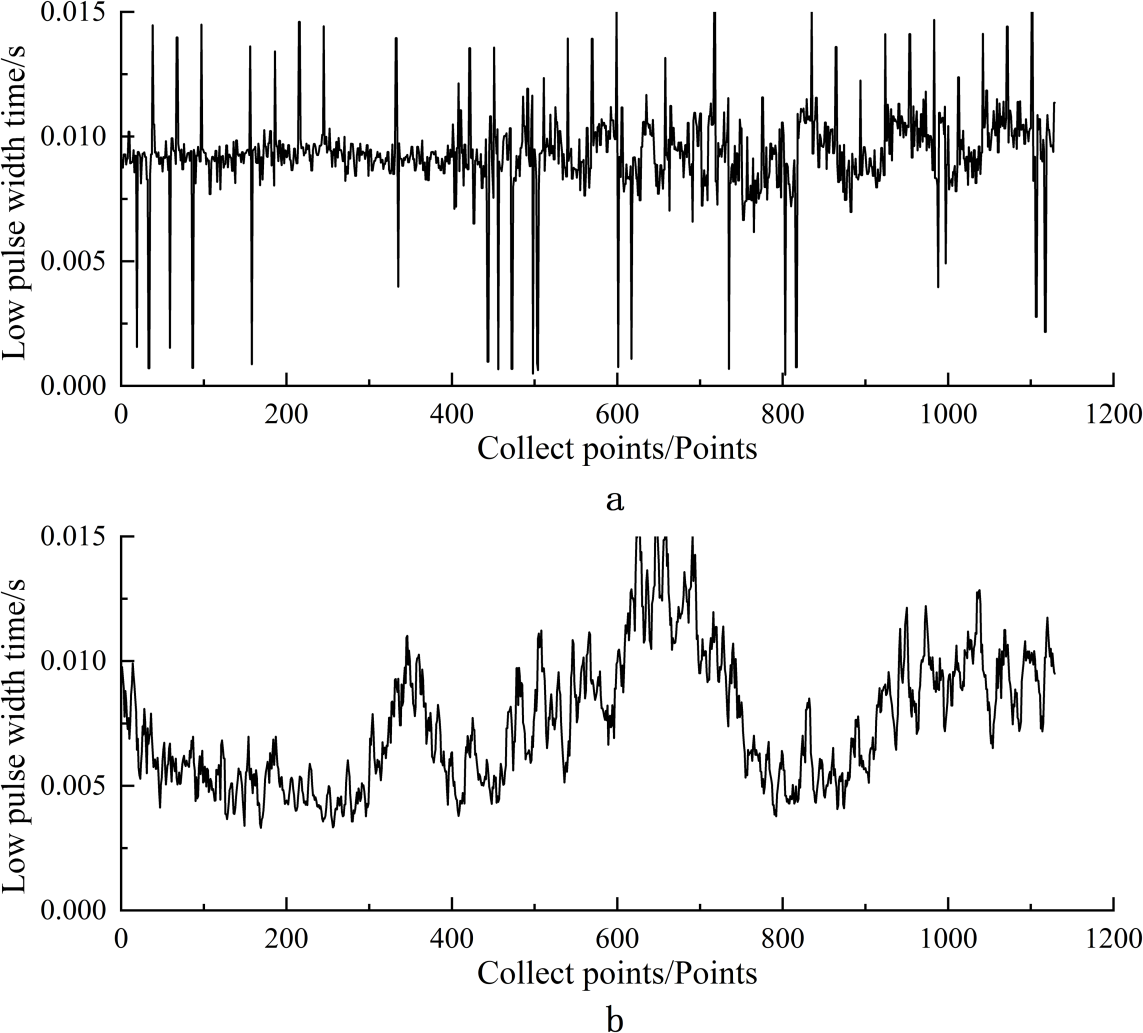
Temporal width of low pulse captured before process **(A)** and after processing **(B)**.

A 380V variable-frequency motor drives the experimental bench, which leads to electromagnetic interference on the sensor signals due to the high current and frequent conversion. The system introduces an optocoupler isolation circuit to suppress the noise of the sensor signals. We observe the abnormal sensor data in the indoor experiments and analyze the elevator’s vibration, which causes the kernel to fall from the scraper, some of which blocks the sensors. Moreover, we also observe such abnormal signals in the field experiments due to falling kernels induced by variant travel speeds and the unevenness of the farmland. Therefore, this paper employs a time-domain threshold smoothing interpolation algorithm to preprocess the data collected by the sensors. When the collected raw data arrives at 10, the following data is processed and filtered. The average value of the previous 10 data points is *V_An_*, and the ratio between the following data and the mean data *V_An_* is determined. Considering the signal changes w.r.t. different conditions, we determine the threshold as *V_MAX_* =3 *V_An_* and 3V*_MIN_* = *V_An_*. During threshold filtering, data exceeding the threshold range are replaced by smoothed interpolation as *V_n_ = V_An_*, while data within the threshold range are retained.


(2) $$V_{n}=\{\begin{array}{ll} V_{An} & \left(V_{n}\leq V_{MIN}\right)\\ V_{n} & \left(V_{MIN}\leq V_{n}\leq V_{MAX}\right)\\ V_{An} & \left(V_{n}\geq V_{MAX}\right) \end{array}$$

where, n>10 , and $V_{n}$ represents the original value of each data processing, $V_{MIN}$ is the minimum value of the threshold range, and $V_{MAX}$ is the maximum value of the threshold range. As observed in **Figure 7A**, the original signal is severely affected by noise interference, making it difficult for the system to identify the proper signal and collect information normally. On the contrary, **Figure 7B** shows the signal after preprocessing and observes that the interference effect is greatly reduced. At the same time, the signal-to-noise ratio has enhanced, promising adequate data information.

#### 3.1.3 Model training results and analysis

**Figure 8:**
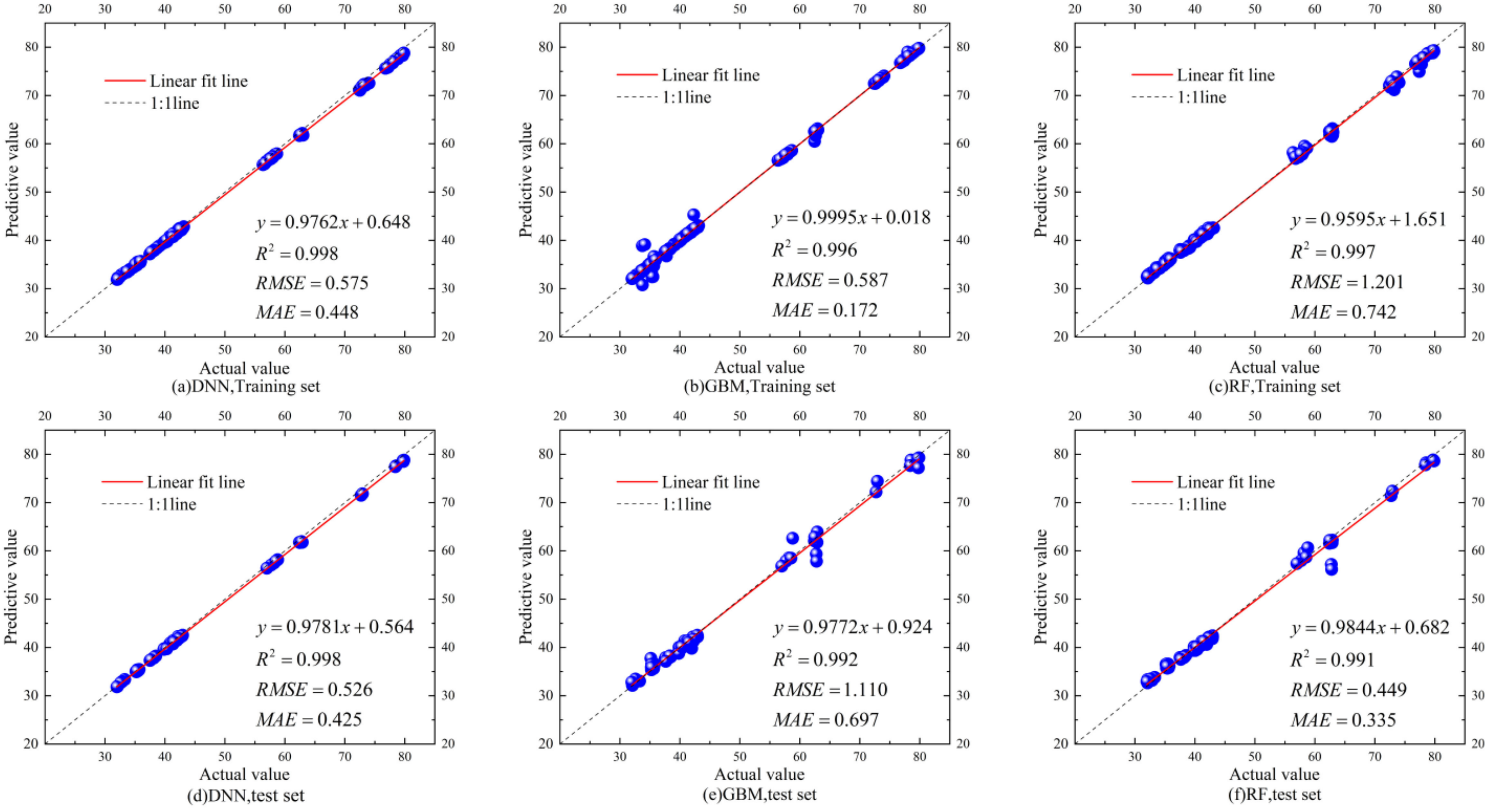
Training and testing of corn yield predictions based on different models with the composite scenario dataset for validating the model effectiveness. **(A)** Performance of DNN with training set, **(B)** performance of GBM with training set, **(C)** performance of RF with training set, **(D)** performance of DNN with test set, **(E)** performance of GBM with test set, and **(F)** performance of RF with test set.

A total of 724 data sets were collected in the experiment, of which 700 sets are valid. Among these datasets, 400 are for compound situation sets and 100 sets for each of the three feeding levels. The data are divided into training and test sets by a ratio of 4:1. We arrived at a DNN model structure with five hidden layers through multiple training and parameter optimizations. The first layer contained 1,024 neurons; half of the neuron number is used in the subsequent layer. The ReLU activation function is also used to enhance the model’s non-linear representation capability, while Dropout is employed to prevent overfitting. The Adam optimizer, with a learning rate of 0.01, is used. The GBM model is built with 100 weak learners, while the RF model also has 100 decision trees with a maximum depth of 3. The loss function utilizes the mean squared error (MSE) running with 300 epochs. **Figure 8** presents the predictive performance of these three yield prediction models derived from the compound dataset. As analyzed, the DNN model achieves the highest R² value of 0.998 for both training and testing sets. It also has stable RMSE and MAE without significant fluctuations. The GBM model exhibits the best fit in the training set; however, the model results in outliers, considerable fluctuations, and lower accuracy with the RMSE higher than 1 for the test set. The RF model performs similarly to the GBM model. Although the predicted results best fit the test set, the results exhibit outliers and considerable variability and lead to a poor RMSE greater than 1 in the training set.

**Figure 9:**
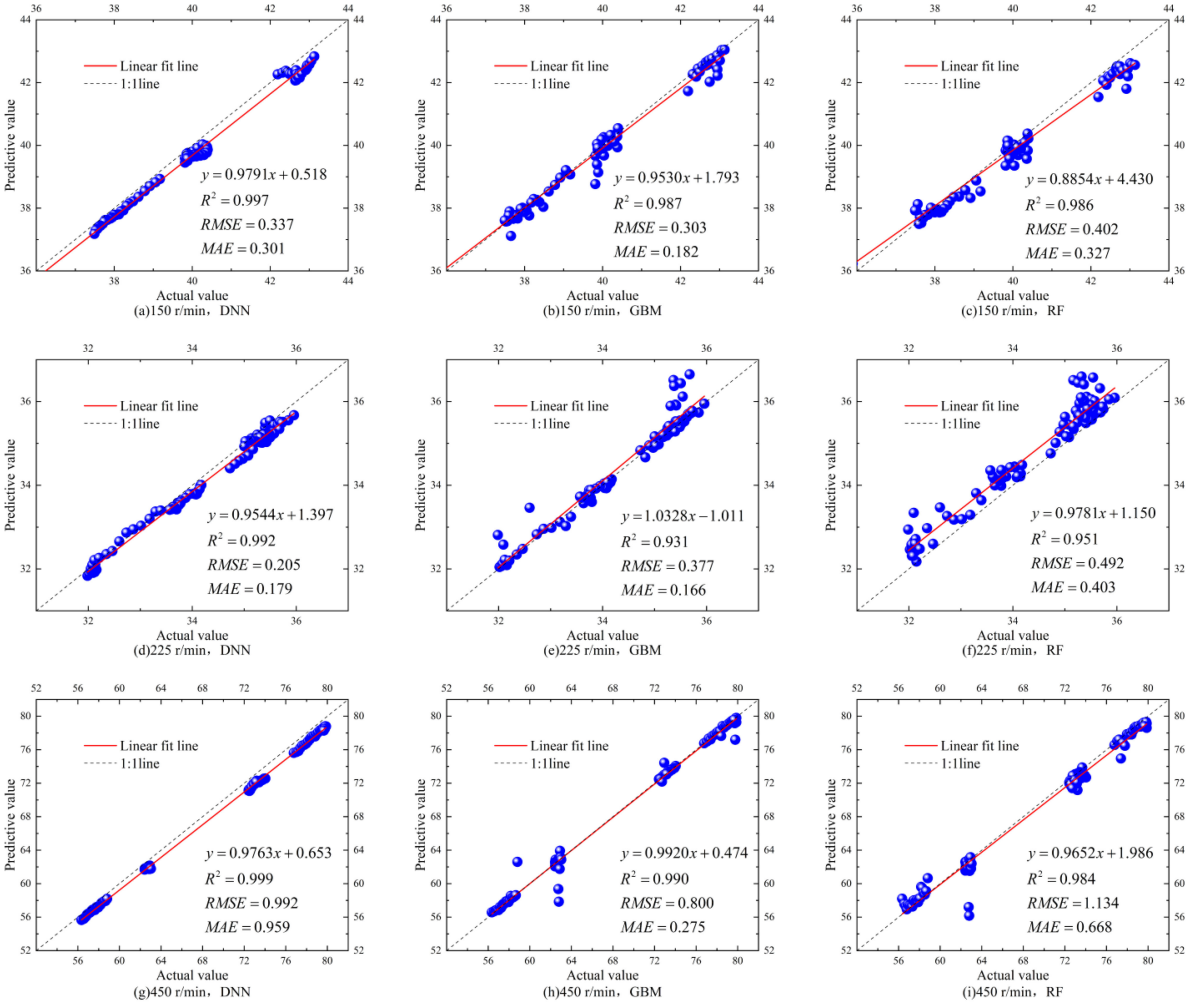
Training and testing of corn yield predictions based on different models under different working conditions for validating the model effectiveness. **(A)** Performance of DNN at 150 r/min, **(B)** performance of GBM at 150 r/min, **(C)** performance of RF at 150 r/min, **(D)** performance at 225 r/min, **(E)** performance of GBM at 225 r/min, **(F)** performance of RF at 225 r/min, **(G)** performance of DNN at 450 r/min, **(H)** performance of GBM at 450 r/min, and **(I)** performance of RF at 450 r/min.

**Figure 9** illustrates the performance of each algorithmic model in predicting corn yields under various working conditions, aiming to determine the model proper for various corn stacking patterns comprehensively. The analysis shows that under low feeding conditions (150 r/min), the DNN model achieves the best fit, with the highest R² value of 0.997. The RF model has the worst fit, with the lowest R² of 0.986, while the GBM model’s performance is intermediate. Under medium feeding conditions (225 r/min), both the GBM and RF models show improved fit but with a higher RMSE, indicating increased error. The GBM model has the most volatility, with an R² of 0.931, while the RF model displays signs of overfitting. The DNN model performs well overall, with the highest R² of 0.992 and considerably low RMSE and MAE. Under high feeding conditions (450 r/min), although the GBM model’s fit is relatively good, it contains numerous poor fitting points. The DNN model demonstrates the highest stability, achieving an R² of 0.999, indicating superior predictive accuracy. In contrast, the RF model performs the worst, with a RMSE greater than 1. In summary, the DNN model consistently predicts the yield with the highest R² values under three working conditions, exhibits the most robust fitting capability, and stably produces low RMSE and MAE. Therefore, we propose the DNN-based prediction model to forecast the corn yields.

### 3.2 Field experiments validation

#### 3.2.1 Design of field experiments

Field experiments for monitoring the corn harvester’s yield were performed to validate the developed system and test the prediction models. These tests were taken in mid-November 2023 in Nongqiao Town, Jiamusi City, Heilongjiang Province. The weather during the experiments was sunny, with temperatures ranging from -11°C to -3°C and a moderate southwest wind at Beaufort scale 3. Given that the crop was snow-covered, daytime melting increased the moisture content of the kernels. Such a condition potentially led to drum clogging and impeded harvesting operations. Consequently, the study carried out the tests at night.

**Figure 10:**
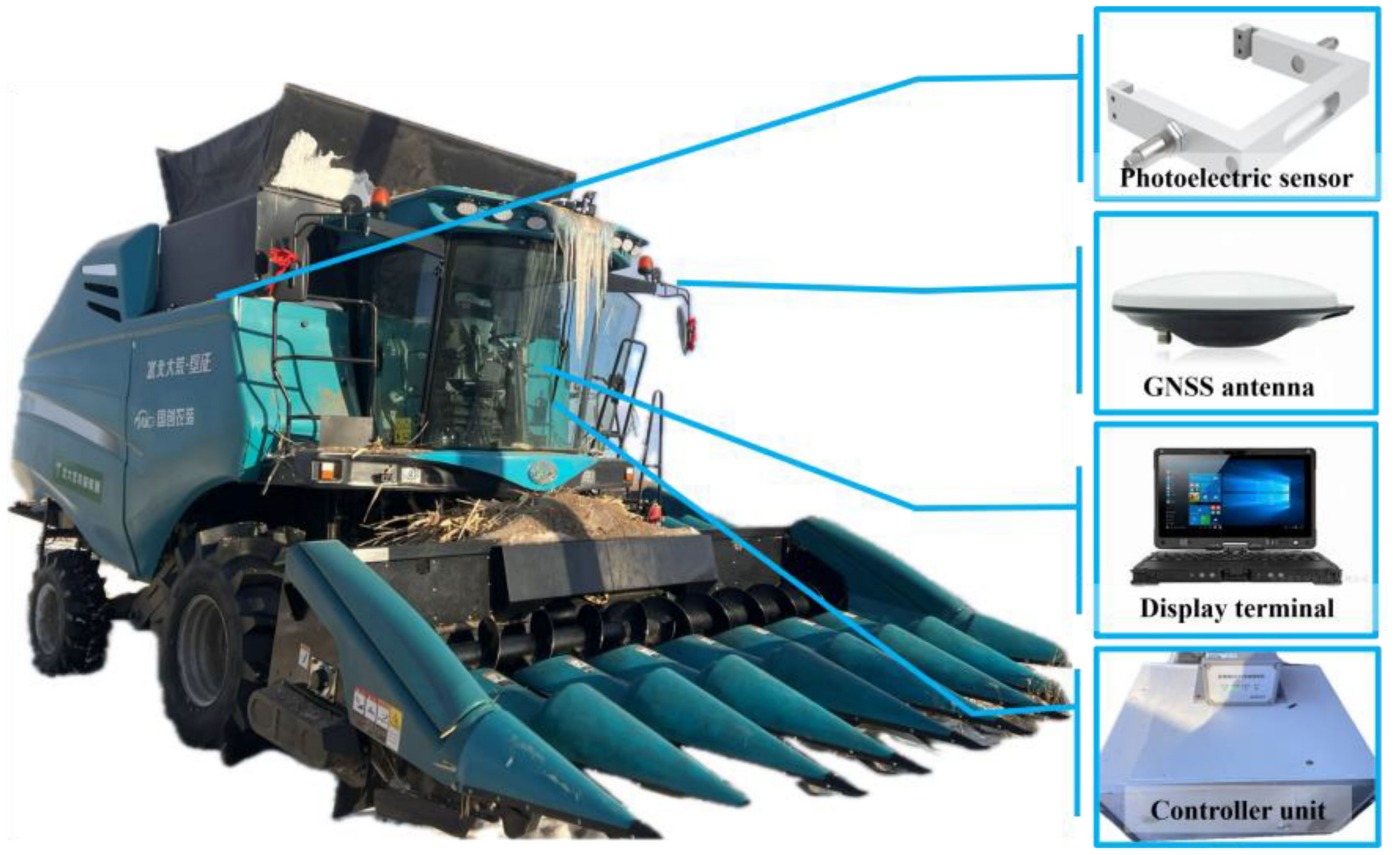
Installation diagram of the system components.

The study installed the system and performed the field experiment on a 4YL-8 corn combine harvester designed and fabricated by the Innovation Centre for Agricultural Machinery and Equipment. **Table 2** presents the harvester’s geometric and working parameters. **Figure 10** demonstrates the installation positions of all the necessary components of the corn yield monitoring system. The thru-beam photoelectric sensor were mounted on a bracket and installed near the top of the harvester’s elevator. The GNSS antenna was mounted on top of the harvester’s side-view mirror. The display terminal, comprising a rugged computer and a controller unit for signal receiving and processing, was placed in the cab for real-time monitoring.

**Table 2:** Related parameters of 4YL-8 corn combine harvester.

Item	Unit	Value
Type	/	4YL-8(8568)
Appearance size (length * width * height)	mm	7850*3540*3950
Number of working rows	/	8
Row spacing	mm	650
Working width	mm	5420
Operating speed	km/h	0-12
Maximum volume of granary	L	7500
Grain unloading method	/	High-level horizontal unloading grain
Driving form	/	Four-wheel drive

#### 3.2.2 Field experiments results and analysis

Before the field experiment, the harvester’s parameters in **Table 2** were set in the yield monitoring system. For the bulk density value in the system, the study collected corn in the field and measured the bulk density, which then replaced the value for the indoor experiment, aiming to eliminate the difference due to corn variety and moisture content, promising a more accurate yield prediction. During the experiment, the system collected the height of the corn pile on scrapers and harvester positions in real-time. The system predicted the instantaneous flow rate and the cumulative yield value accordingly. The actual yield value of the harvester was periodically measured using a platform scale. The study employs and analyzes the relative error between the predicted and the actual yield value by the following equation.


(3) $$E_{r}=\left|\frac{M_{p}-M_{r}}{M_{r}}\times 100\%\right|$$

where, $E_{r}$ is the relative error, $M_{p}$ is the system’s prediction of corn yield, and $M_{r}$ is the actual corn yield.

As presented in **Table 3**, field data are imported into three models to predict the yield, including DNN, GBM, and RF. The results show that the DNN-based model has a maximum relative error of 2.29% in yield prediction, with an average accuracy of 97.85%. The GBM-based model has a maximum relative error of 3.81%, with an average accuracy of 97.8%. The RF-based model demonstrates the highest maximum relative error at 4.26%, with an average accuracy of 96.45%. Although the average accuracy for the DNN and GBM models is similar, the DNN model exhibits notably better stability. The field experimental results denote that the proposed DNN model can reliably predict corn kernel yield during harvesting operations.

**Table 3:** Comparison of yield prediction in field experiments of each model.

Experiment	Medel	Actual yield value(kg)	Predicted yield value(kg)	Relative error(%)
1 2 3	DNN	3488.86 6028.60 5301.98	3414.55 5890.55 5193.82	2.13 2.29 2.04
1 2 3	GBM	3488.86 6028.60 5301.98	3355.93 5979.16 5406.43	3.81 0.82 1.97
1 2 3	RF	3488.86 6028.60 5301.98	3576.43 6263.11 5527.84	2.51 3.89 4.26

## 4 Conclusion

This study developed an advanced yield monitoring system for corn harvesters by exploring photoelectric sensing technology and the characteristics of corn accumulation in the elevator. The system achieved real-time monitoring of harvester operation, yield prediction, and data synchronization. EDEM discrete element simulations were used to observe the accumulation and pattern formation of corn kernels as they ascend in the elevator under different feeding conditions. The simulations highlighted significant variations in kernel morphology, indicating the need for a more sophisticated yield prediction model rather than relying on simple geometric formulas. The study developed an indoor test rig and trained three different machine learning models—Deep Neural Network (DNN), Gradient Boosting Machine (GBM), and Random Forest (RF)—using a composite dataset. These models were validated by operating the auxiliary elevator at various speeds (150 r/min, 225 r/min, and 450 r/min) under different feeding conditions. The DNN-based model demonstrated the best overall performance and stability. Field experiments confirmed the proposed system’s high prediction accuracy.

## Data Availability

The original contributions presented in the study are included in the article/supplementary material, further inquiries can be directed to the corresponding author/s.
